# Mechanism-Independent Manipulation of Single-Wall Carbon Nanotubes with Atomic Force Microscopy Tip

**DOI:** 10.3390/nano10081494

**Published:** 2020-07-30

**Authors:** Dianming Ju, Ying Zhang, Rui Li, Shuang Liu, Longhai Li, Haitao Chen

**Affiliations:** College of Engineering, Northeast Agricultural University, Harbin 150030, China; judianming1995@outlook.com (D.J.); zhangying0604@163.com (Y.Z.); lilh0303@163.com (R.L.); liushuang@neau.edu.cn (S.L.)

**Keywords:** atomic force microscopy, single-wall carbon nanotubes, differentiation evolution algorithm, manipulation trajectory

## Abstract

Atomic force microscopy (AFM) based nanomanipulation can align the orientation and position of individual carbon nanotubes accurately. However, the flexible deformation during the tip manipulation modifies the original shape of these nanotubes, which could affect its electrical properties and reduce the accuracy of AFM nanomanipulation. Thus, we developed a protocol for searching the synergistic parameter combinations to push single-wall carbon nanotubes (SWCNTs) to maintain their original shape after manipulation as far as possible, without requiring the sample physical properties and the tip-manipulation mechanisms. In the protocol, from a vast search space of manipulating parameters, the differential evolution (DE) algorithm was used to identify the optimal combinations of three parameters rapidly with the DE algorithm and the feedback of the length ratio of SWCNTs before and after manipulation. After optimizing the scale factor F and crossover probability *Cr*, the values *F* = 0.4 and *Cr* = 0.6 were used, and the ratio could reach 0.95 within 5–7 iterations. A parameter region with a higher length ratio was also studied to supply arbitrary pushing parameter combinations for individual manipulation demand. The optimal pushing parameter combination reduces the manipulation trajectory and the tip abrasion, thereby significantly improving the efficiency of tip manipulation for nanowire materials. The protocol for searching the best parameter combinations used in this study can also be extended to manipulate other one-dimensional nanomaterials.

## 1. Introduction

Because of their high intrinsic carrier mobility, conductivity, and mechanical flexibility [1,2,3], carbon nanotubes (CNTs) were considered as the next material to be integrated into biosensors [4], nanoswitches [5], flexible circuits [6], and field-effect transistors (FETs). However, the individual CNT-based devices require the precise control of the placement and shape of the CNT. Although several methods have been developed for the array bulk of CNTs, such as the dielectrophoresis technique [7], the orientation of CNTs could be controlled by dielectrophoresis force during the CNTs depositing the substrate surface in the liquid. Moreover, the chemical vapor deposition technique [8,9] is another method used to grow CNTs, which could directly align CNTs during the CNT growth process. However, for individual CNTs, the atomic force microscopy (AFM) [10] cantilever tip-based manipulation is more suitable for controlling the shape and placement of CNTs than the methods mentioned above, which could not control the selected CNT collection amount of aligned CNTs accurately.

AFM tip-based nanomanipulation has been intensively studied in the past decade, helping us to fabricate nanodevices and nanostructures or conduct studies for improving our fundamental understanding of the material physical and chemical properties [11,12,13,14,15,16,17,18,19]. However, the AFM tip cannot perform operations of imaging and manipulation simultaneously. Moreover, scanning-manipulating-scanning reduces efficiency and effectiveness, which hinders AFM tip manipulation [20,21]. Thus, some researchers have developed feedback systems based on the manipulating forces concerning technical difficulties [22,23,24], such as the augmented reality system [25,26,27], which could monitor real-time changes of the nanoenvironment during the manipulating process by analyzing the interaction forces among the tip, substrate, and manipulating target. Some AFM tip control algorithms and drift-compensation methods were also used to push nanoparticles or nanorods [28,29,30,31,32,33,34]. 

For flexible one-dimensional nanomaterials similar to single-wall carbon nanotubes (SWCNTs), a parallel pushing vector manipulating method [33,35] was developed to translate and rotate the flexible nanowires. In this method, the tip-manipulating path was optimized by the finite element method. The CNT bending process was described with a continuum beam-bending model with the clamped−clamped boundary constraints. The research group also designed an automated manipulation system with a pushing trajectory designed automatically by algorithmic programs. Moreover, some researchers have studied a geometric model to control the deforming CNT length and bending angle based on continuum mechanics to design a manipulation path and prevent the CNTs from buckling deformation [36]. However, the original shape of CNTs is challenging to maintain after the pushing operation because the large aspect ratio makes the following manipulation path difficult to predetermine to assure the designed CNTs shape after manipulating. Moreover, the deformation of CNTs will affect its electrical properties [37,38,39]. These problems brought challenges for nanowires manipulation. 

In this study, a multipoint-pushing method with a parallel path was used to push the SWCNTs. The appropriate pushing parameter combinations must be optimized to control the over shape and position accurately. Therefore, the differential evolution (DE) algorithm [40] was introduced to search for the best operating parameter combinations from the parameter spaces to maintain the SWCNTs removal integral. The DE is a population-based stochastic search method for global optimization to solve large-scale combinatorial optimization problems. 

**Figure 1:**
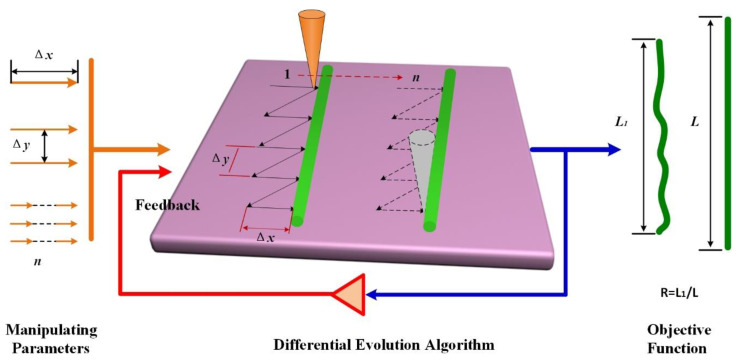
Schematic of the manipulation system. The diagram depicts the tip pushing carbon nanotubes (CNTs) technique loop used for manipulating parameter optimization based on the differential evolution (DE) algorithm and three pushing parameters. Pushing distance Δ*x*, the interval between the adjacent pushing path Δ*y*, and pushing step *n* were fed into the manipulating system. The length ratio of CNT before and after pushing operation was the objective function used to suggest new parameters in the next iteration. The optimization process continues until reaching the desired state.

As shown in Figure 1, the operating parameter combination of the proposed strategy includes three pushing parameters, namely, the pushing distance ∆*x,* the interval between the adjacent pushing path ∆*y*, and the pushing step *n*. The parameter ∆*x* denotes the tip travel distance perpendicular to the axis of CNT, while the interval between the adjacent pushing path ∆*y* is the gap distance between the adjacent tip travel paths. The parameter *n* is the number of repetitions of the tip traveling perpendicular to the axis of CNT. The initial value of the parameters is fed into the DE searching system to optimize the best operating parameter combinations with the feedback of the length ratio *R* of SWCNTs before and after pushing operation. The experimental results indicate that only 5–7 iterations are required to obtain an optimal operating parameter combination, where the length ratio could reach 0.95. With this technique, the CNTs could be transferred with its original shape and the precise position control without optimizing each parameter. Moreover, the pushing path design could prevent the AFM tip from being worn. The method does not require any specific knowledge of the manipulating mechanism and the physical properties of the nanomaterial. 

## 2. Materials and Methods

### 2.1. Manipulating Scheme

The first iteration of the manipulating scheme started with the implementation of four parallel experiments. Moreover, the parameter combinations for these pushing experiments were generated randomly within a large parameter spaces. After manipulating, the shape of CNTs may have differed from the original shape. Therefore, we used the length ratio of CNTs before and after pushing as the system objective function to evaluate the parameter combinations for the results. For the first iteration, the experimental results could not match our expectation and provide an appropriate parameter combination; thus, the DE search algorithm was used in this study to suggest new combinations for the subsequent iterations. By comparing the length ratio values of the first iteration and new iteration, the new parameter combinations with higher values were generated, corresponding to an improved experimental result. Furthermore, the information was fed back to the search algorithm for the subsequent iteration. Subsequently, the search loop continued until no change through iterations occurred in the objective function, which means that the CNTs’ shape after manipulating reached the best state. This approach expended only a few feedback iterations to obtain the best pushing operation parameter combinations without requiring any specific knowledge of the manipulating mechanism and the physical properties of the nanomaterial.

### 2.2. DE Algorithm

**Figure 2:**
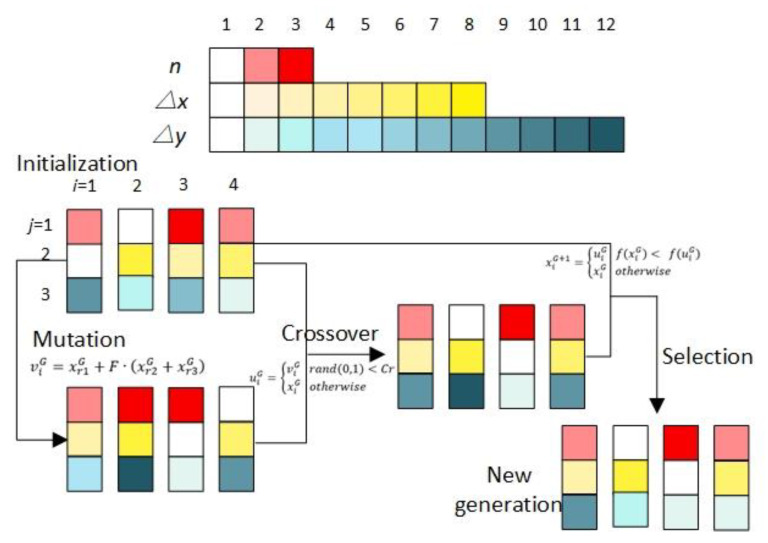
A schematic illustration of DE algorithm.

The stochastic search method, DE algorithm, including initialization, mutation, crossover, and selection, was used for finding the optimal parameter combination to push SWCNTs with precise shape and position control, as shown in Figure 2. In the searching procedures, three parameters, namely, the pushing distance ∆*x*, the interval between the adjacent pushing path ∆*y*, and the pushing step *n*, were used to describe the manipulating condition. Furthermore, the length ratio of CNTs before and after pushing was used as the objective function. 

#### 2.2.1. Initialization

Four parallel experiments were implemented simultaneously, and the initialized parameter combinations $x_{i}^{G}$ (*G* ≥ 0, *i* = 1, 2, 3, 4) were generated from each parameter space, where *G* is the *G*-th iteration, and *i* corresponds to the label of the parallel experiment. Subsequently, the experiments were performed with the parameter combinations $x_{i}^{G}.$ Moreover, the objective function $R\left(\;x_{i}^{G}\;\right)=L_{1}/L$ is the length ratio of CNTs before and after pushing, where *L*_1_ is the length of CNT after pushing, and *L* is the length before pushing. 

#### 2.2.2. Mutation

Once initialized, the DE algorithm starts mutating and recombines the parameter combinations to produce new combinations using: $v_{i}^{G}=x_{r1}^{G}+F\cdot \left(x_{r2}^{G}+x_{r3}^{G}\right),$ where $x_{r1}^{G},\;x_{r2}^{G},\;\mathrm{and}\;x_{r3}^{G}$ correspond to the three different parameters selected from the $x_{i}^{G}$ matrix other than $x_{i}^{G}\;\left(G\geq 0\right)$. *F* is the scale factor, which is a real number between 0 and 1, which is used for controlling the mutation probability in the mutation operator. In our research, the effect of *F* value on the manipulating method was studied, and the best *F* value was optimized from three values, *F* = 0.3, *F* = 0.4, and *F* = 0.5. 

#### 2.2.3. Crossover

A binomial crossover was used in the DE algorithm to increase the parameter diversity by comparing the randomly generated value with the crossover probability *Cr*. This step created new combinations out of the initial combinations of $x_{i}^{G}$ and the mutation combinations $v_{i}^{G}$ using the following equation.

$$u_{i}^{G}=\{\begin{array}{c} v_{i}^{G}\;\;\;rand\left(0,1\right)<Cr\;\;\\ x_{i}^{G}\;\;\;otherwise\;\;\;\;\;\;\;\;\;\;\;\; \end{array}$$

In the equation, the function *rand*(0, 1) generates a random real number between 0 and 1, and the crossover probability *Cr* determines the number of components in $u_{i}^{G}$ out from $x_{i}^{G}$ to ensure the heritage from the last iteration. The definite value of *Cr* ranges from 0 to 1. In this study, the value of *Cr* was obtained by selecting the best value from five values, 0.3, 0.4, 0.5, 0.6, and 0.7.

#### 2.2.4. Selection

The selection was the final step of the searching algorithm, which determined the parameter combinations for the next iteration by comparing the objective function using $x_{i}^{G}\;$ and $u_{i}^{G}$ as in the following equation.

$$x_{i}^{G+1}=\{\begin{array}{c} u_{i}^{G}\;\;\;f\left(x_{i}^{G}\right)<\;f\left(u_{i}^{G}\right)\;\;\\ x_{i}^{G}\;\;\;otherwise\;\;\;\;\;\;\;\;\;\;\;\;\; \end{array}$$

Once the new parameter combination $x_{i}^{G+1}$ was created, the search loop repeated until the objective function achieved a particular threshold, or the objective function had no significant difference with the last iteration. The final parameter combination was the optimal solution. 

### 2.3. AFM Experiments

The AFM (Dimension Icon, Bruker, Santa Barbara, CA, USA) was used to characterize and manipulate a cantilever probe (TESP, Bruker, Santa Barbara, CA, USA). The spring constant of the rectangular cantilever, the resonant frequency, and the tip radius were approximately 42 N/m, 320 kHz, and 8 nm, respectively. Moreover, the AFM images were obtained on tapping mode, and the pushing operation was implemented under contact mode; both operations were performed in an atmospheric environment. 

During scanning, 512 × 512 sample points were used to obtain high-resolution AFM images. The manipulating tip speed was set to 30 nm/s. The manipulating time of a single CNT was up to the manipulating parameters and length of the sample. SWCNT sample was prepared by a standard chemical vapor deposition process. The substrate corresponds to a silicon chip with several hundreds of nanometer thermal oxide on its surface. A 50 μL well-dispersed SWCNT suspension, diluted with an ultrasonic technique, was dropped on the Si substrate surface, and the suspension solvent was evaporated in a dry oven. The length of CNTs used in our experiments, measured with AFM, ranged from 2 to 7 um, and the diameters ranged from 2 to 4 nm. The lateral signal of a position-sensitive detector was acquired with Data Acquisition Card for the tip pushing samples. Moreover, the frictional force between the CNTs and the Si substrate was calculated [24,26,31] to 17 ± 2 nN for the AFM tip pushing distance from 100 nm to 1 um.

## 3. Results and Discussion

### 3.1. Parameters Space Optimization

**Figure 3:**
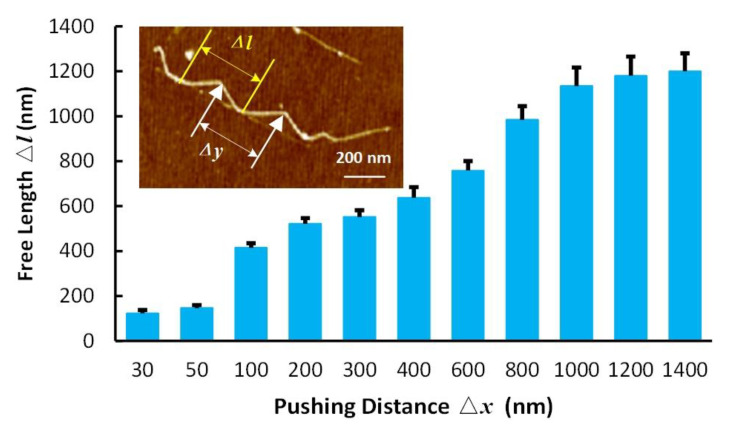
Carbon nanotube (CNT) bending test by atomic force microscopy (AFM) pushing operation with a single path. The bar chart describes the free length Δ*l* under pushing distance Δ*x* used in our study. The inlet image is the AFM image illustrated the free length and the interval between adjacent pushing path Δ*y.*

The AFM tip pushing operation would cause the bending behavior of CNT due to the flexibility of nanowires. As shown in Figure 3, the free length Δ*l*, which is the deformation length of CNT with a single pushing path, should be higher than the interval between the adjacent pushing path Δ*y.* Otherwise, part of the CNT would be stayed at the original location rather than transferred to another position, during the manipulating process. In this study, with a pushing distance of more than 1400 nm, the pre-experiment results showed that the length radio *R* was less than 0.5. After considering these pre-experiment results and the manipulating efficiency, the pushing distance spaces for 30 nm, 50 nm, 100 nm, 200 nm, 300 nm, 400 nm, 600 nm, 700 nm, 800 nm, 1000 nm, 1200 nm, and 1400 nm were found. The free length with different pushing distances was obtained with the AFM tip pushing experiments on the Si substrate, as shown in Figure 3. Note that the free length increment reduced after a pushing distance higher than 1000 nm. Therefore, the maximum value of Δ*y* was set to 1000 nm. The interval between the adjacent pushing path spaces included 50 nm, 100 nm, 200 nm, 300 nm, 400 nm, 600 nm, 800 nm, and 1000 nm. The pushing step spaces number included 1, 2, and 3. 

### 3.2. Intrinsic Parameters of DE Optimization

In the DE search algorithm, except for the manipulating parameters, the intrinsic constant of the scale factor *F* and the crossover probability *Cr* also affected the optimization result by affecting the convergence speed and search space. The high scale factor would increase the mutation probability of the parameters, whereas the low value maintains most parameter combinations, similar to the values in the previous iteration. Moreover, the crossover probability determines the searching space; high *Cr* value improved the local search ability but could lead to premature convergence. For different applications for different intrinsic parameters were considered. In this study, the intrinsic parameters were optimized from the spaces of *F* = 0.3, 0.4, and 0.5, *Cr* = 0.3, 0.4, 0.5, 0.6, and 0.7 with the feedback of the length ratio *R*, which a higher *R*-value means that the shape of CNTs after manipulation was similar to the original shape before manipulating. Moreover, the straight CNTs were selected in our experiments, and the parameter combination was optimized to avoid the deformation of straight CNTs during manipulation. The manipulating parameter values in parameter combinations were selected from the parameter spaces according to the DE algorithm.

#### 3.2.1. F Value Optimization

**Figure 4:**
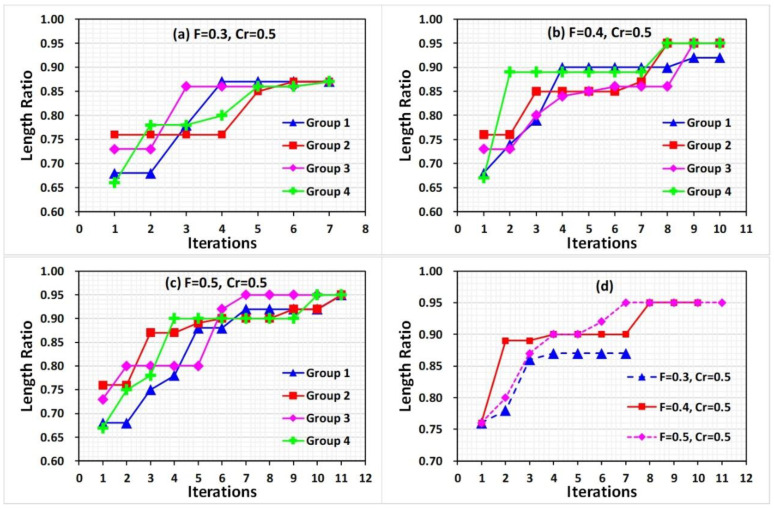
Differential evolution based on the length ratio in feedback control for pushing CNT operation with (**a**) *F* = 0.3, (**b**) *F* = 0.4, and (**c**) *F* = 0.5. The peak length ratio value in each step iteration of every set is listed in (**d**). In each iteration, four groups of combinations were studied.

The first iteration started from a random generation of manipulating parameters from the parameter spaces. For comparison purposes, *Cr* = 0.5 and the same starting parameter combinations were used in this section. However, with different F values, the parameter combinations generated in the mutation and crossover for the next iteration may differ. The parameter combinations are listed in Tables S1–S3 in Supplementary Information. Figure 4a–c show the experimental results, where the length ratio values using the starting parameter combinations ranged from 0.66 to 0.76. After several searching iterations, the results indicate that the length ratio curves presented an increasing trend along with the iterations. 

The optimization converged to its best value throughout the process. Specifically, with *F* = 0.3, the curves approach the value 0.87 within seven iterations, which is a local optimization because a low *F* value reduced the diversity of the pushing parameters in the next iteration, leading to premature convergence. With a higher *F* value, the variance of the pushing parameters increased the chances to search away from local optima. Thus, with *F* = 0.4 and 0.5, the peak length ratio values reached 0.95 at seven or eight iterations. Moreover, the three group results reach 0.95 in nine iterations with *F* = 0.4; however, one group reaches 0.92. With *F* = 0.5, all group results reached 0.95 in 11 iterations. The curves of the peak value in each iteration step of every set showed improved manipulating results regarding the value of *F* = 0.4 and 0.5, as shown in Figure 4d. However, with *F* = 0.4, faster convergence speed and similar results as with *F* = 0.5 were obtained. Therefore, 0.4 was selected in this study as a scaling factor.

#### 3.2.2. Cr Value Optimization

**Figure 5:**
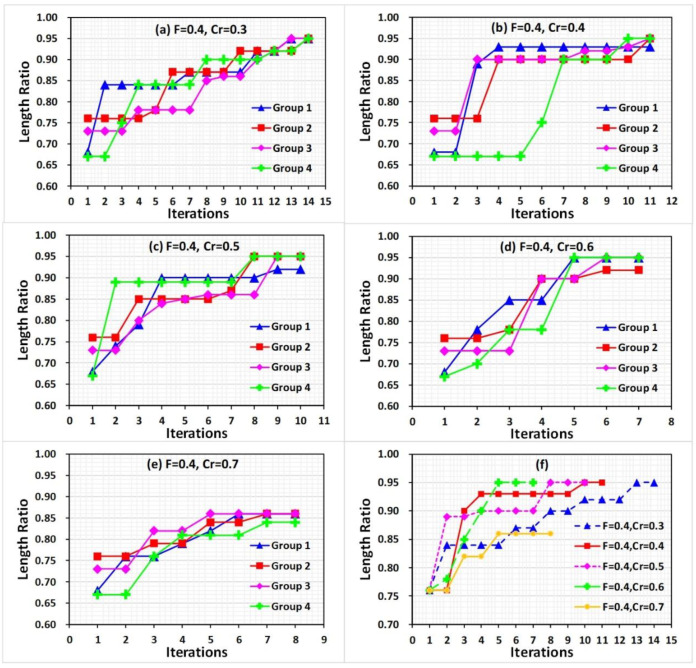
Differential evolution based on the length ratio in feedback control for pushing CNT operation with (**a**) *Cr* = 0.3, (**b**) *Cr* = 0.4, (**c**) *Cr* = 0.5, (**d**) *Cr* = 0.6, and (**e**) *Cr* = 0.7. The peak length ratio value in each step iteration of every set experiments is listed in (**f**). In each iteration, four groups of combinations were studied.

The same starting parameter combinations of previous experiments were used in this section. Five set considering *Cr* = 0.3, 0.4, 0.5, 0.6, and 0.7 were designed to evaluate *Cr*, as shown in Figure 5. For *Cr* = 0.3, considering all groups, the results reach the value of 0.95 in 14 iterations (see Table S4 in Supplementary Information). With an increase in *Cr*, the result curves converge faster. Three result curves reached the value 0.95, and the last group reacheed the value 0.92 with *Cr* = 0.4, 0.5, and 0.6 in 11, 9, and 6 iterations, respectively, as shown in Figure 4b–d (see Tables S2, S5, and S6 in Supplementary Information). However, with the higher *Cr* value of 0.7, the premature convergence phenomenon could not be avoided, and the result curves approached 0.85 in seven iterations, causing local optimization (see Table S7 in Supplementary Information). Comparing the best result in each iteration step of every set, as shown in Figure 5e, the *Cr* = 0.6 curve achieved the value 0.95. All group result curves in this set converge at the value 0.95. Therefore, 0.6 was selected in our research as the *Cr* value.

### 3.3. Pushing Parameter Combination

**Figure 6:**
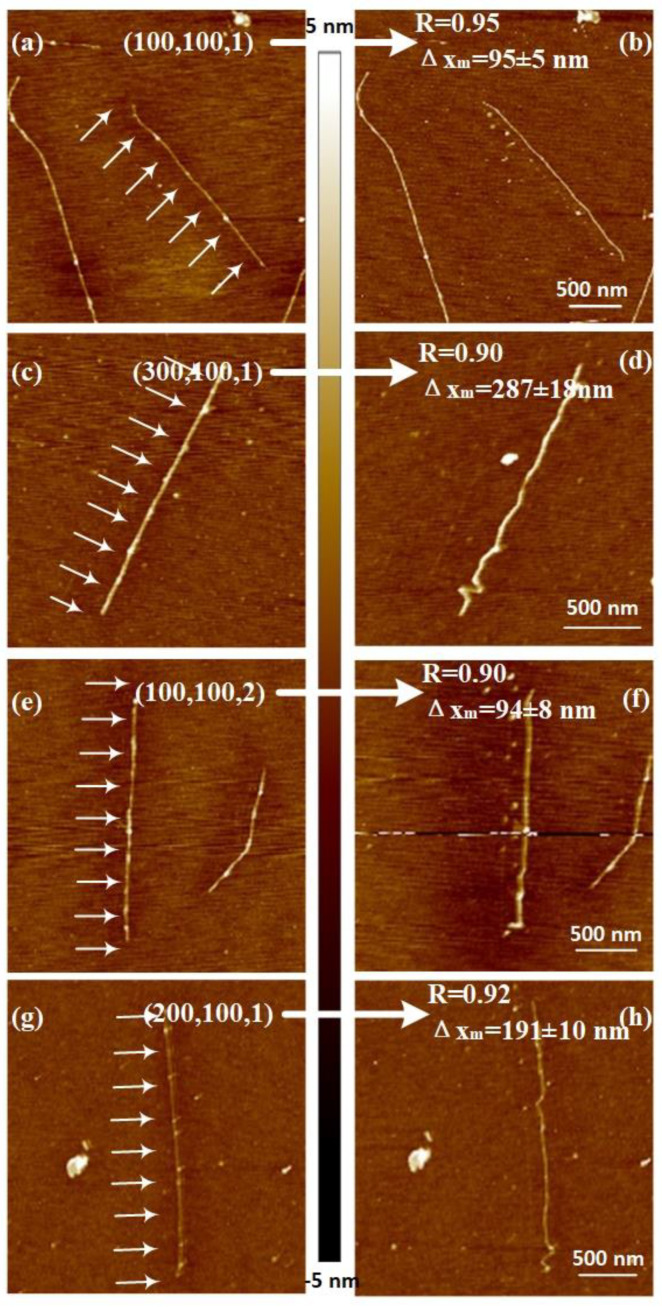
AFM images of CNT before and after pushing operation with different parameter combinations. The arrows in the left column of the AFM images denote the multipoint method and pushing direction. (**a**), (**c**), (**e**) and (**g**) are the AFM images before manipulating operation, (**b**), (**d**), (**f**) and (**h**) are the AFM images after manipulation with parameter combinations respectively. The vectors in the left column are the parameter combinations used in the manipulation, and *R* is the length ratio, Δ*x_m_* is the distance between the position of CNTs before and after manipulating.

Considering the less tip wearing and faster-approaching speed to reach the best experimental result, we used *F* = 0.4 and *Cr* = 0.6 to study the parameter combination for pushing SWCNT with AFM tip on a silicon substrate, as shown in Figure 5d and Table S6. The result curves had an increasing trend with iterations; however, the parameter of the pushing distance Δ*x*, the interval between the adjacent pushing path Δ*y,* and the pushing step *n* decrease until reaching the value 0.95 with the parameter combination (100,100,1), i.e., Δ*x* = 100 nm, Δ*y* = 100 nm, and *n* = 1. Figure 6 shows the AFM images before and after pushing, corresponding to a result above 0.90. The parallel pushing path is denoted in the figure using arrows. Comparing the AFM images before and after manipulating, we found that the CNT with the peak pushing result value of 0.95 was removed, maintaining most of the original straight-line shape. Moreover, other CNTs had some deformation after the pushing operation. The distance between the position of CNTs before and after manipulating Δ*x_m_* was measured, as shown in Figure 6. The measured values all approached the target pushing distance Δ*x* with a 10% error.

For the armchair CNTs, the effects of bending deformation on their electrical transport properties were calculated [41,42,43]. The transmission function was affected strongly when the bending angles were higher than 45°. Moreover, the electrical resistance increased when the bending angles were higher than 45°. However, for zigzag CNTs, the computed conductance was affected significantly when the bending angles were higher than 20°. In the experiments, the parameter combinations with the experimental result of length ratio above 0.90 could be used for armchair CNTs without affecting the electrical transport properties, as the bending angles were less than 45°. Furthermore, the parameter combinations of the experimental result with a length ratio of 0.95 could be used for zigzag CNTs manipulation, as the bending angles were less than 20°. All the pushing parameter combinations having a length ratio value above 0.90 for all the sets of experiments are listed in Table 1. In the table, almost all combinations having a higher value had only one pushing step. Moreover, for a long pushing distance Δ*x* of 100 or 200 nm, the manipulation value of 0.90 or 0.92 was obtained. For a small Δ*x* of 50 or 100 nm, the length ratio *R* could reach 0.95. These results were similar for the interval between the adjacent pushing path Δ*y*. 

**Figure 7:**
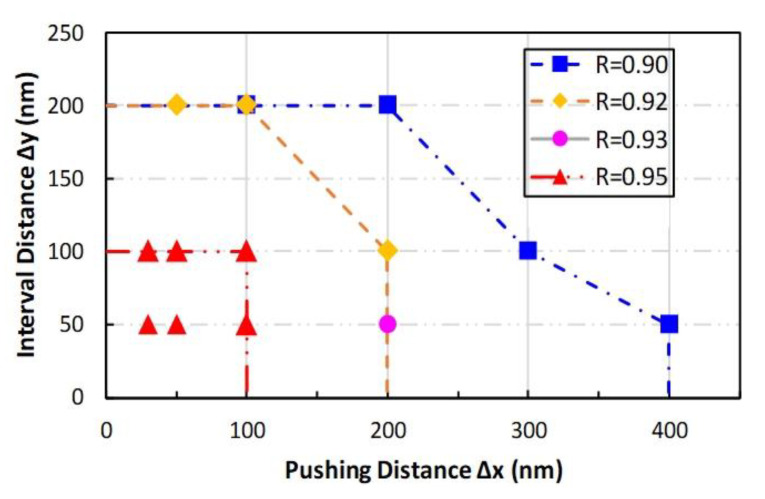
Pushing parameter region with higher length ratio value. The lines were obtained by connecting the same *R*-value combination points.

Figure 7 shows the parameter combinations having only one pushing step in Table 1. The first quadrant of Figure 7 was divided into several regions using different lines, which were formed by connecting the combination coordinate points having the same experimental results. A smaller value of the parameters improving experimental results was obtained in previous experiments. Therefore, if the experiment was performed with a parameter combination selected in the region, the length ratio of CNT before and after pushing would be better than the value with combinations on the boundary line. For example, if the pushing parameters were selected in the area surrounded by the blue line, even though the parameters were not in our parameter spaces, the experimental result of the length ratio would be higher than 0.90. This result was demonstrated because the regions with value *R* > 0.90 were surrounded with the 0.90 line. If a researcher wanted to obtain the best experimental result, the pushing parameters should be selected in the area surrounded by the red line. The parameters region could supply any pushing parameter combination for a user, designing the pushing parameters to achieve an individual demand. 

## 4. Conclusions

The pushing parameters, including the pushing distance, the interval between the adjacent pushing path, and the pushing step, affected the CNT pushing operation substantially. These parameters determine the shape of CNT after the pushing operation. In this study, the DE search algorithm was used to search the optimal pushing parameter combination to avoid the deformation of CNT during manipulation, which could maintain the straight shape of CNT after manipulation similar to before manipulation, as well as the length ratio of CNT before and after pushing operation was set as the objective function. After optimizing the scale factor F and crossover probability *Cr*, the values 0.4 and 0.6 were used, respectively, to search the optimal pushing parameter combination. With only six iterations and 24 pushing experiments, the best pushing parameter combination (100, 100, 1) was obtained, and the experimental result reached the length ratio value of 0.95. 

Moreover, the region of pushing parameters presenting values higher than 0.9 the length ratio value, with a 10% error in the measured pushing distance, was studied to provide arbitrary parameter combinations to researchers, even beyond the analyzed parameter spaces. Furthermore, having a pushing parameters region provides a favorable result for researchers to design the pushing experiments according to their demand. No need for optimizing every parameter saves time and reduces the wearing of AFM tip. Moreover, the fact that the knowledge of the physical properties of samples and the tip manipulation mechanisms are not required is another advantage of this method. Future research will try to characterize the effect of deformation on the physical property of CNTs using the proposed method. We will also try to manipulate other one-dimension nanomaterials, such as the DNA chain. This method could also be extended to other nanomaterial manipulation to assemble nanodevices with precise position and shape control.

## Figures and Tables

**Table 1:** Parameter combinations used in manipulating operation having *R*-value above 0.90 for all experimental data. (Δ*x,* Δ*y, n*) denotes the parameter values in the parameter combination.

	R	0.90	0.92	0.93	0.95
No.					
1		(100, 200, 2)	(50, 200, 1)	(200, 50, 1)	(30, 50, 1)
2		(200, 200, 1)	(100, 200, 1)	-	(30, 100, 1)
3		(300, 100, 1)	(200, 100, 1)	-	(50, 50, 1)
4		(400, 50, 1)	-	-	(50, 100, 1)
5		(100,100,2)	-	-	(100, 100, 1)
6		-	-	-	(100, 50, 1)

## Supplementary Materials

The supplementary materials are available online at https://www.mdpi.com/2079-4991/10/8/1494/s1. Table S1: *F* = 0.3, *Cr* = 0.5, Table S2: *F* = 0.4, *Cr* = 0.5, Table S3: *F* = 0.5, *Cr* = 0.5, Table S4: *F* = 0.4, *Cr* = 0.3, Table S5: *F* = 0.4, *Cr* = 0.4, Table S6: *F* = 0.4, *Cr* = 0.6, Table S7: *F* = 0.4, *Cr* = 0.7.
