# Supplementary material for: Mechanism-Independent Manipulation of Single-Wall Carbon Nanotubes with Atomic Force Microscopy Tip
Source: Nanomaterials (Basel). 2020 Jul 30;10(8):1494. doi: 10.3390/nano10081494 (PMC7466456; doi:10.3390/nano10081494)
Supplement: Supplementary file 1 [file nanomaterials-10-01494-s001.pdf]

Supplementary Material

Mechanism-Independent Manipulation of Single-Wall Carbon Nanotubes with Atomic Force Microscopy Tip

Dianming Ju, Ying Zhang, Rui Li, Shuang Liu, Longhai Li * and Haitao Chen *

College of Engineering, Northeast Agricultural University, Harbin 150030, China; judianming1995@outlook.com (D.J.), zhangying0604@163.com (Y.Z.); lilh0303@163.com (R.L.); liushuang@neau.edu.cn (S.L.)

* Correspondence: lilonghai@neau.edu.cn (L.L.); htchen@neau.edu.cn (H.C.); Tel.: +86-0451-5519-1757 (L.L.); +86-0451-5519-0081 (H.C.)

Table S1: F = 0.3, Cr = 0.5

Iteration	Combination	Step Length nm	Step Gap nm	Step Time	Ratio
1	1	1000	600	1	0.68
	2	400	200	2	0.76
	3	300	300	3	0.73
	4	600	400	2	0.67
2	1	600	400	2	0.66
	2	400	200	2	0.76
	3	600	300	3	0.64
	4	600	300	1	0.78
	1	1000	600	1	0.68
	2	400	200	2	0.76
	3	300	300	3	0.73
	4	600	300	1	0.78
3	1	600	300	1	0.78
	2	400	200	3	0.69
	3	300	300	1	0.86
	4	600	300	1	0.78
	1	600	300	1	0.78
	2	400	200	2	0.76
	3	300	300	1	0.86
	4	600	300	1	0.78
4	1	400	200	1	0.87
	2	400	200	2	0.76

	3	600	300	1	0.78
	4	300	200	2	0.80
	1	400	200	1	0.87
	2	400	200	2	0.76
	3	300	300	1	0.86
	4	300	200	2	0.80
5	1	300	200	2	0.80
	2	400	300	1	0.85
	3	300	200	2	0.80
	4	300	300	1	0.86
	1	400	200	1	0.87
	2	400	300	1	0.85
	3	300	300	1	0.86
	4	300	300	1	0.86
6	1	400	300	1	0.85
	2	400	200	1	0.87
	3	300	300	1	0.86
	4	300	300	1	0.86
	1	400	200	1	0.87
	2	400	200	1	0.87
	3	300	300	1	0.86
	4	300	300	1	0.86
7	1	400	200	1	0.87
	2	300	300	1	0.86
	3	400	200	1	0.87
	4	400	200	1	0.87
	1	400	200	1	0.87
	2	400	200	1	0.87
	3	400	200	1	0.87
	4	400	200	1	0.87

Table S2: F = 0.4, Cr = 0.5

Iteration	Combination	Step Length nm	Step Gap nm	Step Time	Ratio
1	1	1000	600	1	0.68
	2	400	200	2	0.76
	3	300	300	3	0.73
	4	600	400	2	0.67
2	1	1000	200	1	0.74
	2	400	200	2	0.76
	3	600	300	2	0.71
	4	100	100	3	0.89
	1	1000	200	1	0.74
	2	400	200	2	0.76

	3	300	300	3	0.73
	4	100	100	3	0.89
3	1	200	300	3	0.79
	2	400	300	1	0.85
	3	300	100	3	0.80
	4	100	200	3	0.85
	1	200	300	3	0.79
	2	400	300	1	0.85
	3	300	100	3	0.80
	4	100	100	3	0.89
4	1	100	200	2	0.90
	2	400	200	3	0.69
	3	300	100	2	0.84
	4	400	200	1	0.87
	1	100	200	2	0.90
	2	400	300	1	0.85
	3	300	100	2	0.84
	4	100	100	3	0.89
5	1	100	200	2	0.90
	2	400	50	3	0.72
	3	400	300	1	0.85
	4	100	100	3	0.89
	1	100	200	2	0.90
	2	400	300	1	0.85
	3	400	300	1	0.85
	4	100	100	3	0.89
6	1	100	200	2	0.90
	2	400	300	1	0.85
	3	200	300	1	0.86
	4	600	300	1	0.78
	1	100	200	2	0.90
	2	400	300	1	0.85
	3	200	300	1	0.86
	4	100	100	3	0.89
7	1	100	200	2	0.90
	2	100	400	1	0.87
	3	200	100	3	0.85
	4	100	400	1	0.87
	1	100	200	2	0.90
	2	100	400	1	0.87
	3	200	300	1	0.86
	4	100	100	3	0.89
8	1	200	400	1	0.84
	2	100	100	1	0.95

	3	200	300	3	0.79
	4	100	100	1	0.95
	1	100	200	2	0.90
	2	100	100	1	0.95
	3	200	300	1	0.86
	4	100	100	1	0.95
9	1	100	200	1	0.92
	2	100	100	1	0.95
	3	100	100	1	0.95
	4	100	100	1	0.95
	1	100	200	1	0.92
	2	100	100	1	0.95
	3	100	100	1	0.95
	4	100	100	1	0.95
10	1	100	200	1	0.92
	2	100	100	1	0.95
	3	100	100	1	0.95
	4	100	100	1	0.95
	1	100	200	1	0.92
	2	100	100	1	0.95
	3	100	100	1	0.95
	4	100	100	1	0.95

Table S3: F **= 0.5,** Cr **= 0.5**

Iteration	Combination	Step Length nm	Step Gap nm	Step Time	Ratio
1	1	1000	600	1	0.68
	2	400	200	2	0.76
	3	300	300	3	0.73
	4	600	400	2	0.67
2	1	1000	600	1	0.68
	2	400	200	2	0.76
	3	300	200	2	0.80
	4	600	100	2	0.75
	1	1000	600	1	0.68
	2	400	200	2	0.76
	3	300	200	2	0.80
	4	600	100	2	0.75
3	1	600	100	2	0.75
	2	400	200	1	0.87
	3	1000	200	1	0.74
	4	600	100	1	0.78
	1	600	100	2	0.75

	2	400	200	1	0.87
	3	300	200	2	0.80
	4	600	100	1	0.78
4	1	600	100	1	0.78
	2	600	200	1	0.78
	3	400	100	2	0.78
	4	200	200	1	0.90
	1	600	100	1	0.78
	2	400	200	1	0.87
	3	300	200	2	0.80
	4	200	200	1	0.90
5	1	300	200	1	0.88
	2	400	100	1	0.89
	3	300	200	2	0.80
	4	200	200	1	0.90
	1	300	200	1	0.88
	2	400	100	1	0.89
	3	300	200	2	0.80
	4	200	200	1	0.90
6	1	300	200	1	0.88
	2	300	100	1	0.90
	3	100	200	1	0.92
	4	400	200	1	0.87
	1	300	200	1	0.88
	2	300	100	1	0.90
	3	100	200	1	0.92
	4	200	200	1	0.90
7	1	50	200	1	0.92
	2	300	100	1	0.90
	3	100	100	1	0.95
	4	200	200	1	0.90
	1	50	200	1	0.92
	2	300	100	1	0.90
	3	100	100	1	0.95
	4	200	200	1	0.90
8	1	100	200	1	0.92
	2	300	100	1	0.90
	3	100	100	1	0.95
	4	300	200	1	0.88
	1	50	200	1	0.92
	2	300	100	1	0.90
	3	100	100	1	0.95
	4	200	200	1	0.90
9	1	300	200	1	0.88

	2	100	200	1	0.92
	3	100	100	1	0.95
	4	200	200	1	0.90
	1	50	200	1	0.92
	2	100	200	1	0.92
	3	100	100	1	0.95
	4	200	200	1	0.90
10	1	200	100	1	0.92
	2	200	100	1	0.92
	3	100	200	1	0.92
	4	100	100	1	0.95
	1	50	200	1	0.92
	2	100	200	1	0.92
	3	100	100	1	0.95
	4	100	100	1	0.95
11	1	100	100	1	0.95
	2	50	100	1	0.95
	3	100	100	1	0.95
	4	100	100	1	0.95
	1	100	100	1	0.95
	2	50	100	1	0.95
	3	100	100	1	0.95
	4	100	100	1	0.95

Table S4: F = 0.4, Cr = 0.3

Iteration	Combination	Step Length nm	Step Gap nm	Step Time	Ratio
1	1	1000	600	1	0.68
	2	400	200	2	0.76
	3	300	300	3	0.73
	4	600	400	2	0.67
2	1	200	400	1	0.84
	2	400	200	2	0.76
	3	300	300	3	0.73
	4	600	400	2	0.67
	1	200	400	1	0.84
	2	400	200	2	0.76
	3	300	300	3	0.73
	4	600	400	2	0.67
3	1	400	400	1	0.79
	2	400	300	3	0.69
	3	300	300	3	0.73
	4	600	400	1	0.75
	1	200	400	1	0.84

	2	400	200	2	0.76
	3	300	300	3	0.73
	4	600	400	1	0.75
4	1	200	400	1	0.84
	2	400	200	2	0.76
	3	600	300	1	0.78
	4	200	400	1	0.84
	1	200	400	1	0.84
	2	400	200	2	0.76
	3	600	300	1	0.78
	4	200	400	1	0.84
5	1	300	400	1	0.81
	2	600	200	1	0.78
	3	600	300	1	0.78
	4	600	400	1	0.75
	1	200	400	1	0.84
	2	600	200	1	0.78
	3	600	300	1	0.78
	4	200	400	1	0.84
6	1	200	400	1	0.84
	2	400	200	1	0.87
	3	600	200	1	0.78
	4	200	400	1	0.84
	1	200	400	1	0.84
	2	400	200	1	0.87
	3	600	300	1	0.78
	4	200	400	1	0.84
7	1	100	400	1	0.87
	2	400	200	1	0.87
	3	600	300	1	0.78
	4	200	400	1	0.84
	1	100	400	1	0.87
	2	400	200	1	0.87
	3	600	300	1	0.78
	4	200	400	1	0.84
8	1	100	400	1	0.87
	2	400	200	1	0.87
	3	400	300	1	0.85
	4	200	200	1	0.90
	1	100	400	1	0.87
	2	400	200	1	0.87
	3	400	300	1	0.85
	4	200	200	1	0.90
9	1	100	400	1	0.87

	2	400	200	1	0.87
	3	300	300	1	0.86
	4	100	400	1	0.87
	1	100	400	1	0.87
	2	400	200	1	0.87
	3	300	300	1	0.86
	4	200	200	1	0.90
10	1	100	400	1	0.87
	2	50	200	1	0.92
	3	300	300	1	0.86
	4	200	200	1	0.90
	1	100	400	1	0.87
	2	50	200	1	0.92
	3	300	300	1	0.86
	4	200	200	1	0.90
11	1	100	200	1	0.92
	2	50	200	1	0.92
	3	300	100	1	0.90
	4	200	200	1	0.90
	1	100	200	1	0.92
	2	50	200	1	0.92
	3	300	100	1	0.90
	4	200	200	1	0.90
12	1	100	200	1	0.92
	2	50	200	1	0.92
	3	200	100	1	0.92
	4	200	100	1	0.92
	1	100	200	1	0.92
	2	50	200	1	0.92
	3	200	100	1	0.92
	4	200	100	1	0.92
13	1	100	100	1	0.95
	2	50	200	1	0.92
	3	30	100	1	0.95
	4	200	100	1	0.92
	1	100	100	1	0.95
	2	50	200	1	0.92
	3	30	100	1	0.95
	4	200	100	1	0.92
14	1	100	100	1	0.95
	2	50	100	1	0.95
	3	200	100	1	0.92
	4	30	100	1	0.95

	1	100	100	1	0.95
	2	50	100	1	0.95
	3	30	100	1	0.95
	4	30	100	1	0.95

Table S5: ***F*** **= 0.4,** ***Cr*** **= 0.4**

Iteration	Combination	Step Length nm	Step Gap nm	Step Time	Ratio
1	1	1000	600	1	0.68
	2	400	200	2	0.76
	3	300	300	3	0.73
	4	600	400	2	0.67
2	1	600	400	1	0.75
	2	400	200	3	0.69
	3	300	100	2	0.84
	4	600	400	2	0.67
	1	600	400	1	0.75
	2	400	200	2	0.76
	3	300	100	2	0.84
	4	600	400	2	0.67
3	1	200	50	2	0.89
	2	400	600	2	0.70
	3	300	100	1	0.90
	4	600	400	2	0.67
	1	200	50	2	0.89
	2	400	200	2	0.76
	3	300	100	1	0.90
	4	600	400	2	0.67
4	1	200	50	1	0.93
	2	400	50	1	0.90
	3	300	100	2	0.84
	4	600	400	2	0.67
	1	200	50	1	0.93
	2	400	50	1	0.90
	3	300	100	1	0.90
	4	600	400	2	0.67
5	1	600	200	1	0.78
	2	400	50	1	0.90
	3	300	200	1	0.88
	4	600	400	2	0.67
	1	200	50	1	0.93
	2	400	50	1	0.90
	3	300	100	1	0.90
	4	600	400	2	0.67

6	1	400	200	1	0.87
	2	400	50	1	0.90
	3	600	400	1	0.75
	4	600	100	2	0.75
	1	200	50	1	0.93
	2	400	50	1	0.90
	3	300	100	1	0.90
	4	600	100	2	0.75
7	1	600	100	2	0.75
	2	400	50	1	0.90
	3	300	100	1	0.90
	4	400	50	1	0.90
	1	200	50	1	0.93
	2	400	50	1	0.90
	3	300	100	1	0.90
	4	400	50	1	0.90
8	1	200	50	1	0.93
	2	400	50	1	0.90
	3	200	100	1	0.92
	4	400	100	1	0.89
	1	200	50	1	0.93
	2	400	50	1	0.90
	3	200	100	1	0.92
	4	400	50	1	0.90
9	1	400	50	1	0.90
	2	400	50	1	0.90
	3	200	100	1	0.92
	4	400	50	1	0.90
	1	200	50	1	0.93
	2	400	50	1	0.90
	3	200	100	1	0.92
	4	400	50	1	0.90
10	1	300	50	1	0.90
	2	400	50	1	0.90
	3	200	50	1	0.93
	4	100	50	1	0.95
	1	200	50	1	0.93
	2	400	50	1	0.90
	3	200	50	1	0.93
	4	100	50	1	0.95
11	1	400	50	1	0.90
	2	100	50	1	0.95
	3	100	50	1	0.95

	4	300	50	1	0.90
	1	200	50	1	0.93
	2	100	50	1	0.95
	3	100	50	1	0.95
	4	100	50	1	0.95

Table S6: *F* = 0.4, *Cr* = 0.6

Iteration	Combination	Step Length nm	Step Gap nm	Step Time	Ratio
1	1	1000	600	1	0.68
	2	400	200	2	0.76
	3	300	300	3	0.73
	4	600	400	2	0.67
2	1	600	200	1	0.78
	2	400	200	2	0.76
	3	1000	600	1	0.68
	4	600	600	1	0.70
	1	600	200	1	0.78
	2	400	200	2	0.76
	3	300	300	3	0.73
	4	600	600	1	0.70
3	1	200	100	3	0.85
	2	400	100	2	0.78
	3	600	600	1	0.70
	4	600	200	1	0.78
	1	200	100	3	0.85
	2	400	100	2	0.78
	3	300	300	3	0.73
	4	600	200	1	0.78
4	1	200	100	3	0.85
	2	100	100	2	0.90
	3	300	100	1	0.90
	4	400	200	2	0.76
	1	200	100	3	0.85
	2	100	100	2	0.90
	3	300	100	1	0.90
	4	600	200	1	0.78
5	1	100	100	1	0.95
	2	300	100	3	0.80
	3	300	100	1	0.90
	4	100	100	1	0.95
	1	100	100	1	0.95
	2	100	100	2	0.90
	3	300	100	1	0.90

	4	100	100	1	0.95
6	1	30	100	1	0.95
	2	200	100	1	0.92
	3	100	100	1	0.95
	4	200	100	1	0.92
	1	100	100	1	0.95
	2	200	100	1	0.92
	3	100	100	1	0.95
	4	100	100	1	0.95
7	1	100	100	1	0.95
	2	200	100	1	0.92
	3	100	100	1	0.95
	4	200	100	1	0.92
	1	100	100	1	0.95
	2	200	100	1	0.92
	3	100	100	1	0.95
	4	100	100	1	0.95

Table S7: F **= 0.4,** Cr **= 0.7**

Iteration	Combination	Step Length nm	Step Gap nm	Step Time	Ratio
1	1	1000	600	1	0.68
	2	400	200	2	0.76
	3	300	300	3	0.73
	4	600	400	2	0.67
2	1	300	400	2	0.76
	2	400	400	3	0.65
	3	1000	600	1	0.68
	4	600	400	2	0.67
	1	300	400	2	0.76
	2	400	200	2	0.76
	3	300	300	3	0.73
	4	600	400	2	0.67
3	1	300	400	2	0.76
	2	400	400	1	0.79
	3	200	300	2	0.82
	4	300	400	2	0.76
	1	300	400	2	0.76
	2	400	400	1	0.79
	3	200	300	2	0.82
	4	300	400	2	0.76
4	1	400	400	1	0.79
	2	400	300	2	0.75
	3	200	400	2	0.79

	4	300	400	1	0.81
	1	400	400	1	0.79
	2	400	400	1	0.79
	3	200	300	2	0.82
	4	300	400	1	0.81
5	1	200	300	2	0.82
	2	200	400	1	0.84
	3	300	300	1	0.86
	4	300	300	2	0.77
	1	200	300	2	0.82
	2	200	400	1	0.84
	3	300	300	1	0.86
	4	300	400	1	0.81
6	1	300	300	1	0.86
	2	200	300	2	0.82
	3	300	400	1	0.81
	4	300	400	2	0.76
	1	300	300	1	0.86
	2	200	400	1	0.84
	3	300	300	1	0.86
	4	300	400	1	0.81
7	1	300	300	1	0.86
	2	300	300	1	0.86
	3	300	400	1	0.81
	4	200	400	1	0.84
	1	300	300	1	0.86
	2	300	300	1	0.86
	3	300	300	1	0.86
	4	200	400	1	0.84
8	1	300	300	1	0.86
	2	300	300	1	0.86
	3	300	300	1	0.86
	4	200	400	1	0.84
	1	300	300	1	0.86
	2	300	300	1	0.86
	3	300	300	1	0.86
	4	200	400	1	0.84
